# Selective Immune Silencing by Targeted TGF-β Agonists

**DOI:** 10.64898/2026.01.19.700410

**Published:** 2026-01-20

**Authors:** Qinli Sun, Masato Ogishi, Hua Jiang, Alison K. Barrett, Hao Yan, Elsa Sola, Jie Zhang, Peng Xiao, Huiyun Lyu, Ahmad Salehi, Qizhi Tang, Tobias V. Lanz, Mark M. Davis, Robert S. Negrin, K. Christopher Garcia

**Affiliations:** 1. Department of Molecular and Cellular Physiology, Stanford University School of Medicine, Stanford, CA, USA.; 2. Institute for Immunity, Transplantation, and Infection, Department of Medicine, Stanford University School of Medicine, Stanford, CA, USA.; 3. Department of Medicine, Division of Blood and Marrow Transplantation and Cellular Therapy, Stanford University School of Medicine, Stanford, CA, USA.; 4. Division of Gastroenterology and Hepatology, Department of Medicine, Stanford University School of Medicine, CA, USA.; 5. Diabetes Center, University of California, San Francisco, CA, USA.; 6. Gladstone Institute-UCSF of Genomic Immunology, University of California, San Francisco, CA, USA.; 7. Donor Network West, San Ramon, CA, USA.; 8. Department of Surgery, University of California, San Francisco, CA, USA.; 9. Division of Immunology and Rheumatology, Department of Medicine, Stanford University School of Medicine, Stanford, CA, USA.; 10. Department of Microbiology and Immunology, Stanford University School of Medicine, Stanford, CA, USA.; 11. Howard Hughes Medical Institute, Stanford University School of Medicine, Stanford, CA, USA.; 12. Parker Institute for Cancer Immunotherapy, San Francisco, CA, USA.; 13. Department of Structural Biology, Stanford University School of Medicine, Stanford, CA, USA.

## Abstract

Depletion of pathogenic T and B cells is a pillar of first-line therapies for inflammatory, autoimmune, and transplantation-related immunological diseases. However, concerns about adverse events, safety in immunocompromised patients, and disease relapse from incomplete depletion, limit clinical utility. Here, we exploit the immunosuppressive properties of Transforming growth factor beta (TGF-β), through selective “silencing” of T and B cells by a targeted TGF-β mimic agonist derived from Helminths. CD4 and CD8 T cell-targeted TGF-β agonists effectively silence antigen-stimulated T cell activation and expansion in mice and human spleen organoids. A mouse CD4 T cell-targeted TGF-β agonist silences antigen-specific antibody responses by reprogramming pro-inflammatory Th1 and T follicular helper cells into quiescent or regulatory T cell phenotypes *in vivo*. A human CD19 B cell-targeted TGF-β agonist silences antibody responses by disrupting differentiation of germinal center B cells into antibody-secreting cells in human spleen organoids. Cell-type-specific targeted TGF-β agonists ameliorate disease activity in multiple mouse models with minimal off-target effects *in vivo*. Thus, cell-selective TGF-β agonism is a versatile therapeutic strategy for precise silencing of immune functions.

Dysregulated adaptive T and B lymphocytes drive immunopathology in a wide range of immune-mediated conditions, including inflammatory, allergic, autoimmune, and transplant-related diseases^1,2^. Pathogenic CD4 T cells promote inflammation, as well as facilitate cytotoxic CD8^+^ T cell and autoantibody-producing B cell responses, which mediate tissue destruction and organ dysfunction^1,2^. Therapeutic strategies that deplete or functionally restrain pathogenic T and B cells have been extensively explored across a range of immunological diseases^3–5^. B–cell–depleting monoclonal antibodies (mAbs), such as the CD20-targeting agent Ocrelizumab, have been used to treat multiple sclerosis^6,7^, and Obinutuzumab has also been evaluated in clinical trials for active lupus nephritis^8^. Recently, CD19-targeted CAR-T cells have demonstrated promising efficacy in clinical trials for a number of autoimmune diseases, including systemic lupus erythematosus and systemic sclerosis^9–11^. T-cell-suppressing agents, such as tacrolimus, have been widely used in preventing rejection of hematopoietic stem cell and organ transplantation^12,13^. T cell-depleting agents, such as CD3-specific antibodies teplizumab and otelixizumab, have demonstrated therapeutic effects in preclinical or clinical studies of acute allograft rejection in renal transplantation and type 1 diabetes^14^.

While valuable, depleting therapies have liabilities. First, the elimination of critical cells of the adaptive immune system renders individuals vulnerable to infections. For instance, broad-based T-cell suppression (e.g., tacrolimus) or depletion (e.g., teplizumab) increases the risk of severe infections, including viral reactivation^15^. Long-term B cell depletion causes hypogammaglobulinemia and carries an elevated risk for severe infections^16,17^. Strategies to selectively deplete or suppress pathogenic immune populations would be ideal to mitigate the risk of infections but are not yet available. Second, T- and B-cell-depletion therapies, such as anti-CD3 mAbs and CD19 CAR-T cell therapies, often cause local and systemic toxicities due to profound cytotoxic responses, including local immune effector cell-associated toxicity syndrome (LICATS), cytokine release syndrome (CRS) and immune effector cell–associated neurotoxicity syndrome (ICANS)^18,19^. These cytotoxicity-associated toxicities often necessitate treatment cessation and ancillary immunosuppression, leading to disease rebound. Lastly, cell depletion induces compensatory hematopoiesis, and repopulated cells can acquire pathogenic phenotypes to drive disease relapse, or even initiate new inflammatory disorders^20–22^. The reemergence of autoantibody-producing clones can occur more than five years after B-cell-depletion-induced disease remission^23^. These challenges warrant the development of alternative approaches for immune cell-type-specific suppression.

Transforming growth factor-β (TGF-β) is a pleiotropic cytokine that broadly regulates both immune and non-immune cells^24^, acting as a general enforcer of immune tolerance and suppressor of inflammation^25,26^. TGF-β plays highly context-dependent roles in T cell biology: it inhibits T cell activation and expansion, promotes peripherally induced regulatory T cell (pTreg) development to enforce tolerance, drives pro-inflammatory type 17 helper CD4^+^ T (Th17) cell differentiation, and supports tissue-resident memory T cell formation^27^. Beyond T cells, TGF-β suppresses B cell proliferation and activation while stimulating IgA class-switch recombination^28^. Despite the well-established link between defective TGF-β signaling and heightened inflammation and autoimmunity, its direct effects on mature T and B cell activation and differentiation *in vivo*, and the underlying mechanisms, remain incompletely characterized, as many immunosuppressive effects have historically been attributed to Treg modulation^29^. Moreover, poor drug-like properties, latent homodimer formation, and pro-fibrotic or tumorigenic effects in non-immune cells have severely limited its safe and effective use for *in vivo* immunosuppression^24^.

We have harnessed the immunosuppressive properties of TGF-β to develop immune cell–targeted TGF-β agonists by fusing a low-affinity TGF-β “surrogate agonist” derived from helminths to T cell– or B cell–specific targeting modules. This approach enables selective activation of TGF-β signaling in T and B cells, particularly antigen-activated clones, achieving precise immunosuppression while minimizing off-target effects. We show that mouse and human CD4 and CD8 T cell–targeted, as well as B cell–targeted, TGF-β agonists selectively suppress activation and expansion of their respective cell types in mouse *in vivo* models and human organoids. T cell–targeted agonists exhibit strong therapeutic efficacy in multiple mouse models of neuroinflammation and transplantation-associated disease. These findings highlight a promising therapeutic strategy for T– and B–cell–driven diseases through selective silencing of disease-reactive lymphocytes using targeted TGF-β agonists.

## Design and characterization of mouse T cell-targeted TGF-β agonists.

**Extended Data Figure 1.**
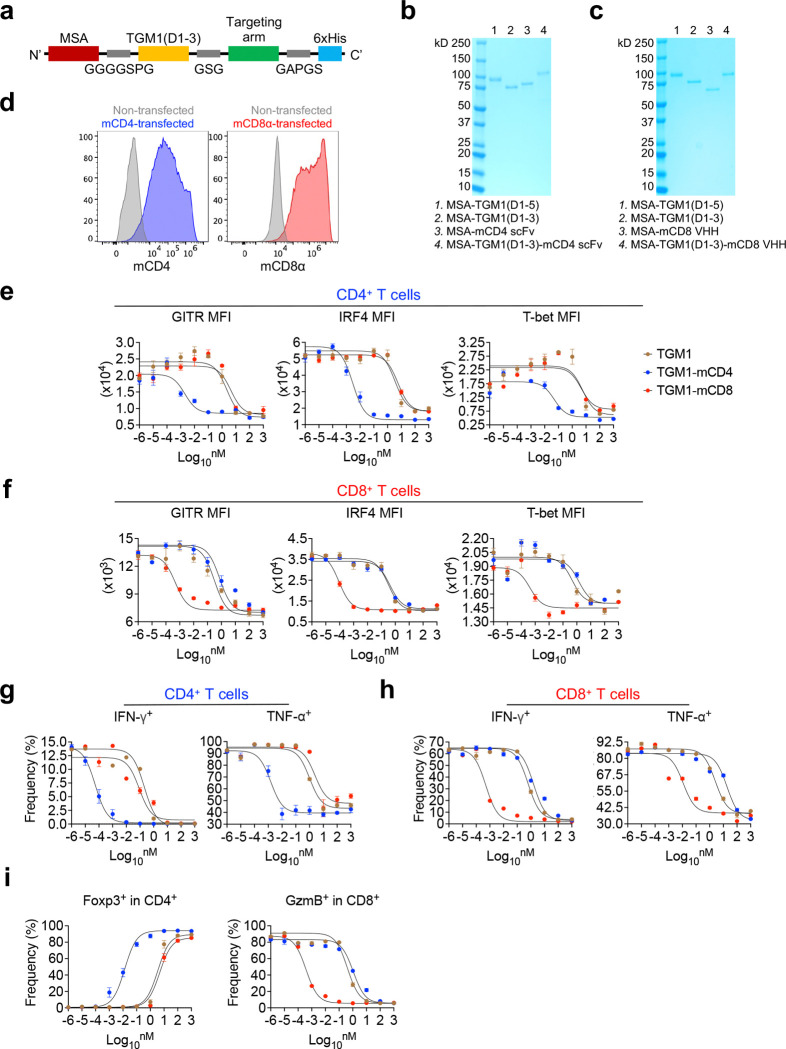
Design and characterization of mouse T cell-targeted TGF-β agonists. **a.** Schematic of the plasmid construct encoding the MSA–TGM1-targeting-arm fusion protein. **b.** SDS–PAGE analysis showing the molecular weight and purity of the indicated recombinant proteins. **c.** SDS–PAGE analysis showing the molecular weight and purity of the indicated recombinant proteins. **d.** Representative flow cytometry plots showing mouse CD4 and CD8α expression in mouse CD4- or CD8α-transduced HEK293 cells. **e.** Dose-dependent curves showing GITR, IRF4, and T-bet expression levels in *in vitro*–cultured CD4 T cells treated with the indicated proteins on day 3. **f.** Dose-dependent curves showing GITR, IRF4, and T-bet expression levels in *in vitro*–cultured CD8 T cells treated with the indicated proteins on day 3. **g.** Dose-dependent curves showing percentages of IFN-γ– and TNF-α–producing cells among *in vitro*–cultured CD4 T cells treated with the indicated proteins on day 3. **h.** Dose-dependent curves showing percentages of IFN-γ– and TNF-α–producing cells among *in vitro*–cultured CD8 T cells treated with the indicated proteins on day 3. **i.** Dose-dependent curves showing Foxp3 cell percentages in *in vitro*-cultured CD4 T cells, and Granzyme B–producing cell percentages in *in vitro*-cultured CD8 T cells treated with the indicated proteins on day 3. Data are presented as mean ± s.e.m.

**Figure 1.**
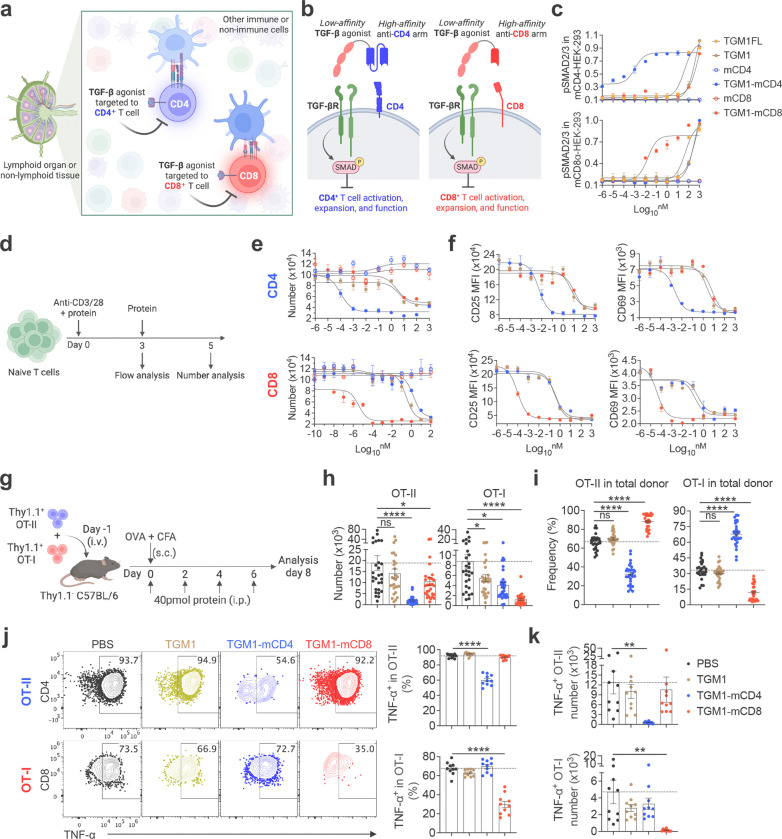
Design and characterization of mouse T cell-targeted TGF-β agonists. **a.** Cartoon showing selective suppression of T cells by T cell-targeted TGF-β agonists *in vivo*. **b.** Cartoons illustrating the design of T cell-targeted TGF-β agonists and their regulation in T cells. **c.** Dose-response curves showing pSMAD2/3 levels in mouse CD4 - or CD8α-transduced HEK293 cells using a pSMAD-induced Secreted Alkaline Phosphatase (SEAP) assay. **d.** Experimental design of mouse T cell *in vitro* culture. **e.** Dose-dependent curves showing *in vitro* cultured CD4 or CD8 T cell numbers treated with the indicated proteins on day 5. **f.** Dose-dependent curves showing CD25 and CD69 expression levels in *in vitro* cultured CD4 or CD8 T cells treated with the indicated proteins on day 3. **g.** Experimental design of naïve OT-II and OT-I cell transfer, OVA immunization, and protein administration. **h.** Quantification of OT-II and OT-I cell numbers in DLNs from the indicated mice. **i.** Quantification of the proportion of OT-II or OT-I cells within total donor cells in DLNs from the indicated mice. **j.** Representative flow cytometry plots and quantification of TNF-α–producing cell percentages in OT-II or OT-I cells from DLNs of the indicated mice. **k.** Quantification of TNF-α–producing cell numbers in OT-II or OT-I cells from DLNs of the indicated mice. Data are presented as mean ± s.e.m. The statistics were obtained by one-way ANOVA coupled with Dunnett’s multiple-comparisons test **(h-k)**.

Our strategy for designing immune cell–targeted TGF-β agonists was to fuse a low-affinity TGF-β agonist with high-affinity antibody cell–targeting arms, enabling selective TGF-β signaling in targeted cells while reducing off-target effects (Fig. 1a and 1b). The TGF-β agonist used here, TGF-β mimic 1 (TGM1), is a helminth-derived Ig-domain protein that activates SMAD2/3 signaling by engaging TGF-β receptors^30,31^. Full-length helminth TGM1 (TGM1FL) contains five domains: domains 1–3 bind TGF-β receptors with low affinity, whereas domains 4–5 bind CD44 with high affinity to achieve broad-based immunosuppression and facilitate immune evasion in infected hosts^31,32^. To target mouse T cells, we replaced the CD44 targeting domains of TGM1 with a high-affinity mouse CD4-targeting scFv^33^ (TGM1-mCD4) or a high-affinity mouse CD8α-targeting VHH^34^ (TGM1-mCD8) via a flexible GSG linker, enabling selective delivery of TGF-β signaling mediated by the low-affinity TGM1 domains 1–3 (hereafter referred to as TGM1) to CD4 or CD8α T cells while largely sparing other cell types (Fig. 1b and Extended Data Fig. 1a). We further fused the construct to mouse serum albumin (MSA) to improve *in vivo* half-life time. This molecule displayed ideal biochemical properties and high expression levels as a monodisperse recombinant protein expressed from Expi293 cells (Extended Data Fig. 1b and 1c). pSMAD assays in mCD4- or CD8α-transduced HEK-293 cells showed that TGM1-mCD4 and TGM1-mCD8 strongly enhanced SMAD2/3 activation, shifting the dose–response curve ~4 logs and ~3 logs, respectively, compared with TGM1 alone, whereas the targeting arms alone had no effect (Fig. 1c and Extended Data Fig. 1d). These experiments validated the enhanced therapeutic windows of the targeted low-affinity TGM1 agonists conferred by high-affinity targeting.

To validate selective T-cell suppression, naïve CD4 or CD8 T cells were stimulated *in vitro* with anti-CD3/CD28 in the presence of these proteins (Fig. 1d). TGM1-mCD4 and TGM1-mCD8 strongly and selectively suppressed TCR-driven CD4 or CD8 T-cell expansion, respectively, shifting the dose–inhibition cell-number curve ~5–6 logs leftward relative to TGM1 alone or the alternative T-cell–targeted TGM1 construct (Fig. 1e). Additionally, TGM1-mCD4 and TGM1-mCD8 effectively suppressed their respective T cell activation, as shown by leftward shifts in dose–response inhibition curves for CD25, CD69, GITR, IRF4, and T-bet expression, as well as for inflammatory cytokines IFN-γ and TNF-α (Fig. 1f and Extended Data Fig. 1e–h). Furthermore, TGM1-mCD4 effectively promoted Foxp3 Treg induction in CD4 T cells, whereas TGM1-mCD8 effectively suppressed Granzyme B production in CD8 T cells (Extended Data Fig. 1i). Together, these data demonstrate that mouse CD4 - or CD8 T cell-targeted TGM1 selectively and effectively suppresses activation and expansion of the corresponding T cell subset *in vitro*.

## Mouse CD4 and CD8 T cell-targeted TGF-β agonists selectively suppress the corresponding antigen-specific T cell responses in OVA-immunized mice.

**Extended Data Figure 2.**
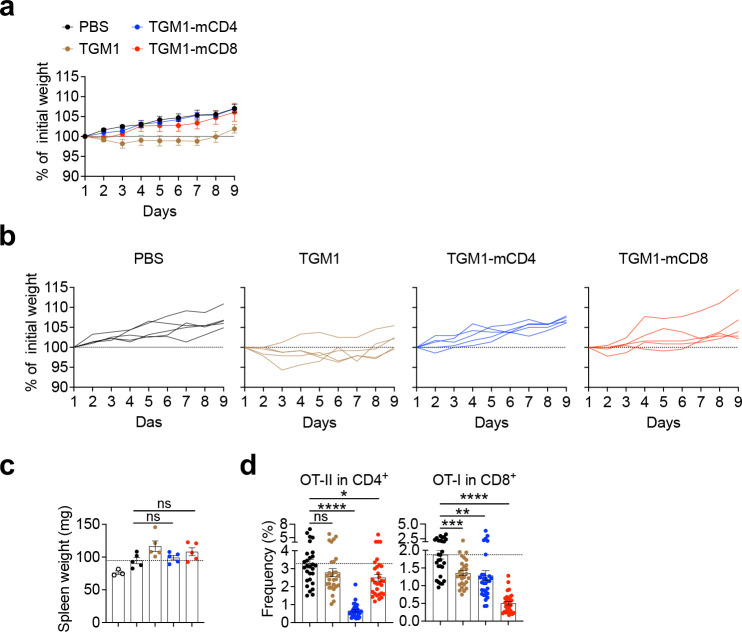
Mouse CD4 or CD8 T cell-targeted TGF-β agonists selectively silence OVA-specific CD4 or CD8 T cell responses, respectively, in OVA-immunized mice. **a.** Mean body weight change curves of mice from the indicated groups. **b.** Individual mouse body weight change curves from the indicated groups. **c.** Quantification of spleen weights from mice in the indicated groups. **d.** Quantification of the proportion of OT-II or OT-I cells within corresponding CD4 or CD8 T cells in DLNs from the indicated mice. Data are presented as mean ± s.e.m. The statistics were obtained by one-way ANOVA coupled with Dunnett’s multiple-comparisons test **(c and d)**.

Next, we assessed the *in vivo* safety and efficacy of TGM1-mCD4 and TGM1-mCD8 on antigen-specific T cell responses. Naïve Thy1.1^+^ CD4 OT-II and CD8 OT-I cells were co-transferred into recipient mice, followed by OVA emulsified in complete Freund’s adjuvant (CFA) to elicit a robust inflammatory response (Fig. 1g). Data showed that mice treated with TGM1-mCD4 or TGM1-mCD8 had body weight and spleen weights comparable to PBS-treated controls, indicating favorable *in vivo* safety and preserved immune homeostasis, whereas low-affinity TGM1 caused modest weight loss (Extended Data Fig. 2a–c). We further analyzed the quantity and phenotype of donor OT-II and OT-I cells. Consistent with their selective targeting, TGM1-mCD4 markedly reduced both the number and frequency of OT-II cells among total CD4 T cells in the draining lymph nodes (DLNs), while TGM1-mCD8 selectively and robustly reduced OT-I cells among total CD8 T cells (Fig. 1h and Extended Data Fig. 2d). Notably, TGM1-mCD4 treatment decreased the proportion of OT-II cells and correspondingly increased OT-I cell frequency among total donor cells, whereas TGM1-mCD8 produced the opposite pattern (Fig. 1i), highlighting their highly selective suppression of the respective T cell populations. Moreover, both TGM1-mCD4 and TGM1-mCD8 specifically and strongly reduced TNF-α–producing cell frequencies and numbers in their respective OT-II and OT-I populations (Fig. 1j and 1k). Together, these results demonstrate that TGM1-mCD4 and TGM1-mCD8 selectively and potently suppress OVA-specific CD4 or CD8 T cell expansion and inflammatory cytokine production *in vivo*, while having minimal effects on the other T cell subsets.

## Phenotypic characterization of antigen-stimulated T and B cells in OVA-immunized mice administered with T cell-targeted TGF-β agonists.

**Extended Data Figure 3.**
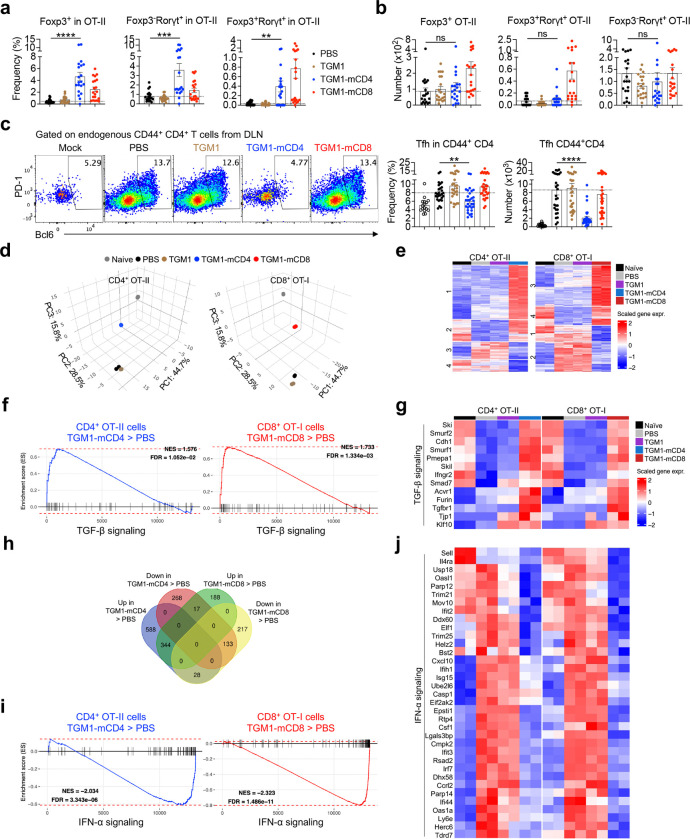
T cell phenotypes in OVA-immunized mice treated with mouse T cell-targeted TGF-β agonists. **a.** Quantification of Foxp3, Foxp3 RORγt, and Foxp3 RORγt OT-II cell frequencies in the DLNs of mice from the indicated groups. **b.** Quantification of Foxp3, Foxp3 RORγt, and Foxp3 RORγt OT-II cell numbers in the DLNs of mice from the indicated groups. **c.** Representative flow cytometry plots and quantification of PD-1 Bcl6 Tfh cell percentages and absolute numbers among endogenous CD4 T cells in the DLNs of the indicated mice. **d.** 3D PCA plots showing the transcriptional profiles of OT-II or OT-I samples across distinct groups. **e.** Heatmaps showing the transcriptional profiles of OT-II or OT-I samples across distinct groups. **f.** GSEA plots showing the enrichment of TGF-β signaling signature genes in the transcriptomes of OT-II or OT-I cells from T cell-targeted TGM1 versus PBS. **g.** Heatmaps showing expression levels of TGF-β signaling feature genes across groups in OT-II or OT-I cells. **h.** Venn diagrams showing overlapping DEGs in OT-II and OT-I cells between T cell-targeted TGM1 and PBS groups. **i.** GSEA plots showing the enrichment of IFN-α signaling signature genes in the transcriptomes of OT-II or OT-I cells from T cell-targeted TGM1 versus PBS. **j.** Heatmaps showing expression levels of IFN-α signaling feature genes across groups in OT-II or OT-I cells. Data are presented as mean ± s.e.m. The statistics were obtained by one-way ANOVA coupled with Dunnett’s multiple-comparisons test **(a-c)**.

**Figure 2.**
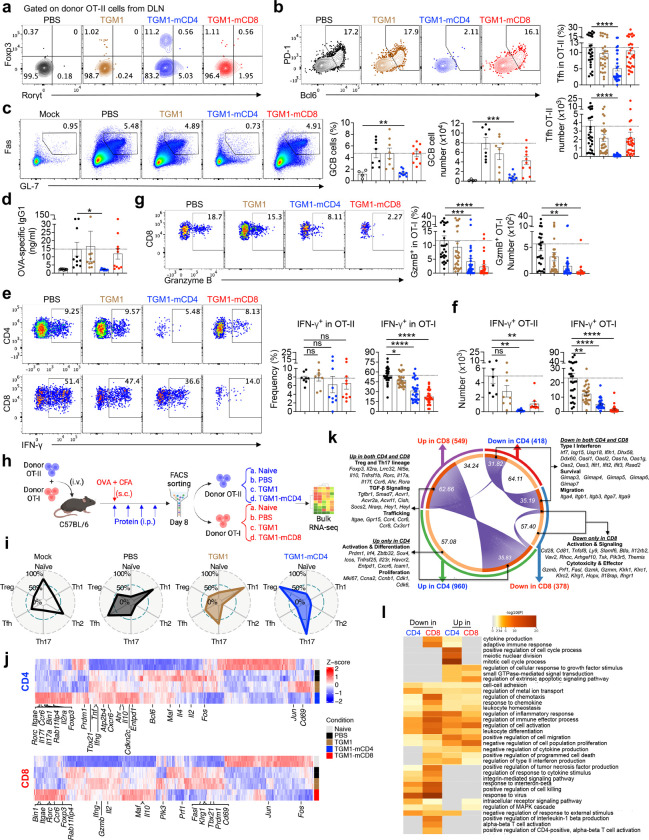
Mouse T cell-targeted TGF-β agonists silence antigen-specific T and B cell responses in OVA-immunized mice. **a.** Representative flow cytometry plots showing Foxp3 and RORγt expression in OT-II cells from DLNs of the indicated mice. **b.** Representative flow cytometry plots and quantification of PD-1^+^Bcl6^+^ Tfh OT-II cell frequencies and numbers from DLNs of the indicated mice. **c.** Representative flow cytometry plots and quantification of Fas GL-7 GCB cell frequencies and numbers from DLNs of the indicated mice. **d.** Serum OVA-specific IgG1 levels in the indicated mice. **e.** Representative flow cytometry plots and quantification of IFN-γ–producing cell percentages in OT-II or OT-I cells from DLNs of the indicated mice. **f.** Quantification of IFN-γ–producing cell numbers in OT-II or OT-I cells from DLNs of the indicated mice. **g.** Representative flow cytometry plots and quantification of Granzyme B–producing cell frequencies and numbers among OT-I cells from DLNs of the indicated mice. **h.** Schematic of the experimental design for bulk RNA-seq sample preparation. **i.** Spider plots showing enrichment of various CD4 T-cell subset signatures in the transcriptomes of OT-II cells from the indicated groups. **j.** Heatmaps showing expression levels of feature genes across groups in OT-II or OT-I cells. **k.** Chord diagram illustrating overlapping genes and highlighted feature genes. **l.** Gene Ontology (GO) pathway analysis showing enriched pathways among upregulated or downregulated DEGs in OT-II or OT-I cells comparing the T-cell-targeted TGM1 and PBS groups. Data are presented as mean ± s.e.m. The statistics were obtained by one-way ANOVA coupled with Dunnett’s multiple-comparisons test **(b-g)**.

We further analyzed the heterogeneous T cell phenotypes, particularly the diverse CD4 T cell subsets, among OVA-specific T cells *in vivo*. Consistent with the established role of TGF-β in promoting CD4 Foxp3 peripherally induced Treg (pTreg) and RORγt type 17 helper (Th17) cell differentiation^35,36^, TGM1-mCD4 modestly increased the frequencies of Foxp3, RORγt Foxp3, and RORγt Foxp3 OT-II cells in the DLNs; however, their absolute numbers were not increased, likely due to inhibition of cell expansion (Fig. 2a, and Extended Data Fig. 3a and 3b). TGM1-mCD8 also modestly increased the frequencies of these OT-II subsets (Fig. 2a, and Extended Data Fig. 3a and 3b), possibly via indirect effects from T cell interactions. These results suggest that while T cell–targeted TGM1 can modestly promote antigen-specific pTreg and Th17 differentiation *in vivo*, it does not generate substantial population sizes. Given the critical role of follicular helper CD4^+^ T (Tfh) cells in mediating B cell responses, we next focused on this population and found that TGM1-mCD4 markedly reduced both the frequency and number of Tfh cells among OT-II and endogenous CD4 T cells in the DLNs, indicating potent suppression of OVA-induced Tfh cell development (Fig. 2b, and Extended Data Fig. 3c). Consistently, germinal center B (GCB) cell differentiation was impaired in DLNs of TGM1-mCD4–treated mice, as shown by markedly reduced frequencies and numbers of Fas GL7 B cells (Fig. 2c). Notably, serum OVA-specific IgG1 levels were markedly decreased (Fig. 2d). Together, these data demonstrate that TGM1-mCD4 potently suppresses antigen-specific Tfh cell development, thereby inhibiting GCB cell formation and antigen-specific antibody production in mice. Additionally, although TGM1-mCD4 did not significantly reduce the frequency of IFN-γ–producing OT-II cells in DLNs, their absolute numbers were markedly decreased (Fig. 2e and 2f). TGM1-mCD8 specifically reduced both the frequency and number of IFN-γ– and Granzyme B–producing OT-I cells, which were modestly suppressed by TGM1-mCD4, likely reflecting reduced CD4 T cell help for CD8 T cells^37^ (Fig. 2e–g). These results suggest that these T cell–targeted agonists suppress type I effector T cell development.

We further analyzed the transcriptomes of these OT-II and OT-I cells via bulk RNA sequencing (Fig. 2h). Principal component analysis (PCA) and gene cluster analyses showed that OT-II and OT-I cells treated with T cell–targeted TGM1s displayed transcriptional profiles distinct from PBS controls and shifted away from a naïve state, indicating induction of a unique cellular program (Fig. 2i, Extended Data Fig. 3d and 3e). In contrast, T cells treated with low-affinity TGM1 closely resembled PBS controls (Fig. 2i, Extended Data Fig. 3d and 3e), underscoring its minimal nonspecific activity and high selectivity and potency conferred by T cell–targeting arms. Gene set enrichment analysis (GSEA) further confirmed that OT-II and OT-I cells treated with T cell–targeted TGM1s exhibited upregulated TGF-β signaling signature genes compared with PBS controls (Extended Data Fig. 3f and 3g). Consistently, GSEA revealed that TGM1-mCD4 potently skewed OT-II cells toward Treg and Th17 transcriptional programs, with strong reductions in Th1 and Tfh signatures (Fig. 2i). Correspondingly, canonical Treg genes (e.g., *Foxp3, Il10, Il2ra, Entpd1*, and *Itgae*) and Th17 genes (e.g., *Rorc, Ahr, Maf, Il17a, Il17f*, and *Ccr6*) were significantly upregulated (Fig. 2j).

Further analysis of differentially expressed genes (DEGs) revealed substantial overlap in transcriptomic changes between CD4 and CD8 T cells. Specifically, 35.58% of TGM1-mCD4–upregulated DEGs in OT-II cells overlapped with 62.66% of TGM1-mCD8–upregulated DEGs in OT-I cells, while 31.82% of downregulated genes in OT-II cells overlapped with 35.19% in OT-I cells (Fig. 2k and Extended Data Fig. 3h). Consistently, DEGs in OT-II and OT-I cells were enriched in numerous overlapping biological pathways (Fig. 2l). Notably, T cell–targeted TGM1s upregulated similar signatures in both OT-II and OT-I cells, associated with Treg and Th17 identity (*Foxp3, Rorc, Ahr, Il10, Il17a, Ccr6*), TGF-β signaling (*Tgfbr1, Smad7, Acvr1, Acvr2a, Acvrl1*), and acquisition of tissue-trafficking properties (*Itgae, Gpr15, Ccr4, Ccr6, Ccr8, Cx3cr1*) (Fig. 2j–l). In contrast, the most prominent gene module consistently downregulated in both OT-II and OT-I cells was the interferon-stimulated gene (ISG) module, including *Irf7, Isg15, Usp18, Ifih1, Dhx58, Ddx60, Oasl*, and Ifit family genes, which are associated with effector and activated T cell states (Fig. 2k, 2l, Extended Data Fig. 3i and 3j). These cells also consistently downregulated GIMAP family members (*Gimap3, Gimap4, Gimap5*), key regulators of T cell survival and homeostasis, as well as integrin family members (*Itga4, Itgb1, Itgb3, Itga7*), which govern T cell migration and tissue interactions (Fig. 2k and 2l).

These agonists also induced subset-specific transcriptional changes. TGM1-mCD4 uniquely upregulated transcription factors (*Prdm1*, *Irf4*, *Zbtb32*) and surface molecules (*Icos*, *Tnfrsf25*, *Cxcr6*, *Icam1*, *Entpd1*) in OT-II cells, which are associated with Treg and Th17 cell activation, function, and migration (Fig. 2j and 2k). It also upregulated genes involved in cell cycle progression and proliferation (*Mki67*, *Ccna2*, *Ccnb1*, *Cdk1*) (Fig. 2k and 2l). These seemingly paradoxical programs likely reflect the heightened plasticity and effector-like state of Treg and Th17 cells. In contrast, TGM1-mCD8 uniquely downregulated in OT-I cells a cytotoxic module associated with cell killing, including *Gzmb*, *Prf1*, and *Fasl*, as well as effector and co-stimulatory genes (*Klrk1*, *Klrg1*, *Cd28*, *Cd81*, *Tnfsf8*, *Il12rb2*) (Fig. 2j–l).

Collectively, these data indicate that T cell–targeted TGM1s program T cells into a distinct transcriptional state biased toward Treg and Th17 signatures, while suppressing Tfh and type I effector programs. This state is further characterized by prominent downregulation of interferon-stimulated, cytotoxic, and activation signatures, consistent with a functionally restrained, quiescent-like state *in vivo*.

## Human CD4 and CD8 T cell-targeted TGF-β agonists selectively silence the corresponding antigen-stimulated T cell responses in human spleen organoids.

**Extended Data Figure 4.**
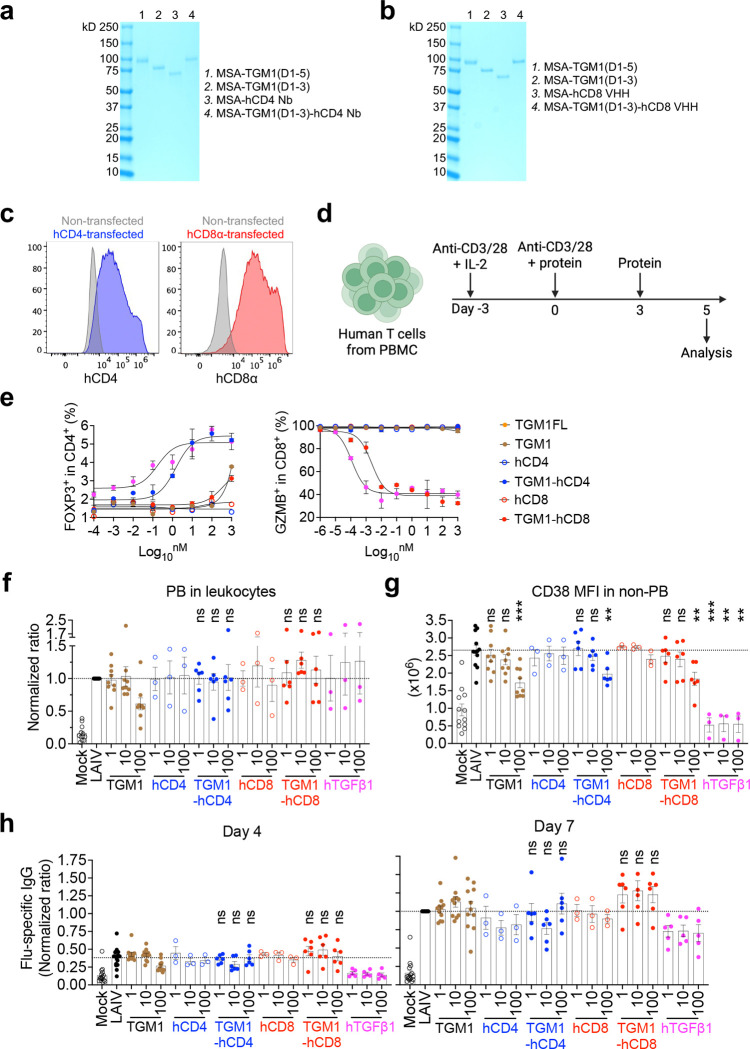
Design and characterization of human T cell-targeted TGF-β agonists. **a.** SDS–PAGE analysis showing the molecular weight and purity of the indicated recombinant proteins. **b.** SDS–PAGE analysis showing the molecular weight and purity of the indicated recombinant proteins. **c.** Representative flow cytometry plots showing human CD4 and CD8α expression in human CD4- or CD8α-transduced HEK293 cells. **d.** Experimental design of human T cell *in vitro* culture. **e.** Dose-dependent curves showing FOXP3 cell percentages in *in vitro*-cultured CD4 T cells, and Granzyme B–producing cell percentages in *in vitro*-cultured CD8 T cells treated with the indicated proteins on day 5. **f.** Quantification of CD38^high^ PB percentages among total leukocytes from the indicated treatment groups. **g.** Quantification of CD38 expression levels in CD38^low^ non-PB from the indicated treatment groups. **h.** Quantification of flu-specific IgG levels in culture supernatants from the indicated treatment groups on days 4 and 7. Data are presented as mean ± s.e.m. The statistics were obtained by one-way ANOVA coupled with Dunnett’s multiple-comparisons test **(f-h)**.

**Figure 3.**
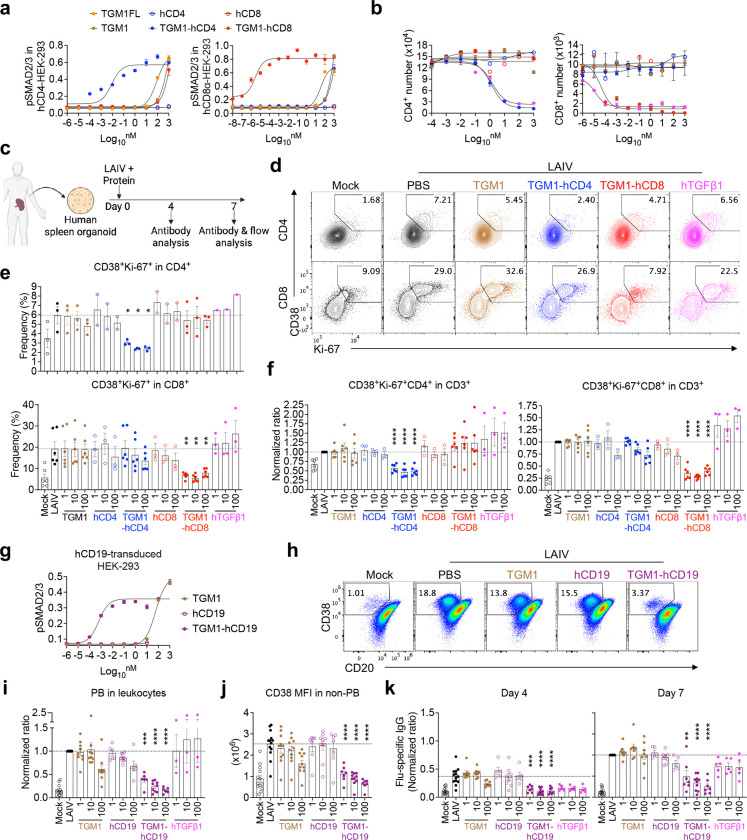
Human T and B cell-targeted TGF-β agonists silence LAIV-induced T and B cell responses in human spleen organoids. **a.** Dose–response curves showing pSMAD2/3 levels in human CD4- or CD8α-transduced HEK293 cells measured using a pSMAD–induced SEAP assay. **b.** Dose-dependent curves showing numbers of *in vitro*–cultured CD4 or CD8 T cells treated with the indicated proteins on day 5. **c.** Schematic of the experimental design for human spleen organoid culture, LAIV stimulation, and protein treatments. **d.** Representative flow cytometry plots showing percentages of CD38 Ki-67 cells in CD4 or CD8 T cells from the indicated treatment groups. **e.** Quantification of CD38 Ki-67 cell percentages in CD4 or CD8 T cells from the indicated treatment groups. **f.** Quantification of CD38 Ki-67 CD4 or CD8 T cell percentages among total CD3 T cells from the indicated treatment groups. **g.** Dose–response curves showing pSMAD2/3 levels in human CD19-transduced HEK293 cells measured using a pSMAD-induced SEAP assay. **h.** Representative flow cytometry plots showing CD38^high^ PB percentages in B cells from the indicated treatment groups. **i.** Quantification of CD38^high^ PB percentages among total leukocytes from the indicated treatment groups. **j.** Quantification of CD38 expression levels in CD38^low^ non-PB from the indicated treatment groups. **k.** Quantification of flu-specific IgG levels in culture supernatants from the indicated treatment groups on days 4 and 7. Data are presented as mean ± s.e.m. The statistics were obtained by one-way ANOVA coupled with Dunnett’s multiple-comparisons test **(e, f, and i-k)**.

We next made human CD4 and CD8 T cell-targeted TGF-β agonists by similarly fusing the low-affinity TGM1 domains 1–3 with a high-affinity human CD4-targeting nanobody^38^ (TGM1-hCD4) and a human CD8α-targeting VHH^39^ (TGM1-hCD8), respectively (Extended Data Fig. 4a and 4b). pSMAD assays in human CD4- or CD8α-transduced HEK293 cells confirmed that TGM1-hCD4 and TGM1-hCD8 shifted the pSMAD2/3 dose–response curve leftward by approximately 4 logs and 8 logs, respectively, compared with low-affinity TGM1 alone, demonstrating their markedly enhanced functional affinity (Fig. 3a and Extended Data Fig. 4c). By culturing CD4 or CD8 T cells from PBMCs of healthy donors with anti-CD3/CD28 stimulation in the presence of these proteins (Extended Data Fig. 4d), we found that TGM1-hCD4 and recombinant human TGF-β1 (hTGFβ1) markedly reduced CD4 T cell numbers, while TGM1-hCD8 and hTGFβ1 potently reduced CD8 T cell numbers in a dose-dependent manner (Fig. 3b). In contrast, low-affinity TGM1, the targeting arms, and the other targeted TGM1 had no effect (Fig. 3b). Moreover, TGM1-hCD4 and hTGFβ1 substantially increased the proportion of FOXP3 CD4 T cells, whereas TGM1-hCD8 and hTGFβ1 strongly reduced Granzyme B–producing CD8 T cells (Extended Data Fig. 4e). These results demonstrate that human CD4 - and CD8-targeted TGM1 selectively and effectively suppress their respective human T cells *in vitro*.

To better mimic *in vivo* conditions, we assessed their efficacy in human spleen organoids stimulated with Live Attenuated Influenza Vaccine (LAIV) (Fig. 3c). TGM1-hCD4 and TGM1-hCD8 selectively and potently suppressed the corresponding LAIV-stimulated CD38 Ki-67 CD4 or CD8 T cells, as reflected by reduced frequencies within both the targeted subsets and total CD3 T cells (Fig. 3d–f). In contrast, hTGF-β1 had no significant effect on these populations, likely due to its broad, non-specific activity and the associated sink effect (Fig. 3d–f). Moreover, plasmablast (PB) frequencies, CD38 expression in non-PB cells, and influenza (flu)–specific IgG levels were unaltered by human T cell–targeted TGM1s at both day 4 and day 7 (Extended Data Fig. 4f–h). These findings demonstrate that TGM1-hCD4 and TGM1-hCD8 specifically suppress antigen-stimulated human CD4 and CD8 T cell activation and proliferation, but are insufficient to suppress B cell responses in human spleen organoids.

## Human B-cell-targeted TGF-β agonist selectively silences antigen-stimulated human B cell activation and antibody production in human spleen organoids.

**Extended Data Figure 5.**
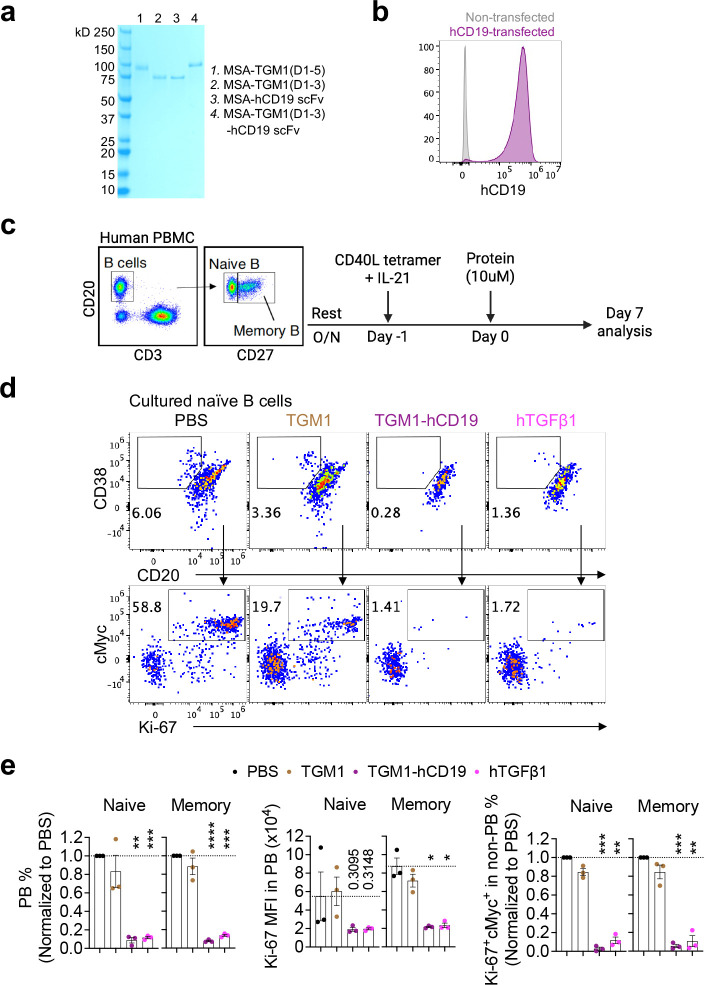
Design and characterization of human B cell-targeted TGF-β agonist. **a.** SDS–PAGE analysis showing the molecular weight and purity of the indicated recombinant proteins. **b.** Representative flow cytometry plots showing human CD19 expression in human CD19-transduced HEK293 cells. **c.** Sorting and stimulation of naïve and memory B cells isolated from PBMCs of healthy donors. **d.** Representative flow cytometry plots of PB development and Ki-67 and c-Myc expression in non-PB cells in sort-purified naïve B cell cultures. **e.** Normalized counts of PBs and their Ki-67 expression, and normalized counts of K-i67 c-Myc non-PB cells. Data are presented as mean ± s.e.m. The statistics were obtained by one-way ANOVA coupled with Dunnett’s multiple-comparisons test **(e)**.

Given the insufficient B-cell silencing by the T cell-targeted TGF-β agonists in human spleen organoids, we developed a human B-cell-targeted TGF-β agonist by fusing the low-affinity TGM1 domains 1–3 to a high-affinity human CD19 scFv^40,41^ (TGM1-hCD19), which induced pSMAD2/3 reporter activity with an approximately five orders of magnitude higher affinity than untargeted TGM1 (Fig. 3g, Extended Data Fig. 5a and 5b). In naïve and memory B cells sorted from healthy donor PBMCs, TGM1-hCD19 or hTGF-β1 inhibited CD20^dim^CD38^high^ PB development by approximately tenfold compared with PBS- or untargeted TGM1-treated cells (Extended Data Fig. 5c–e). Memory B cell-derived PBs treated with TGM1-hCD19 also exhibited significantly lower Ki-67 expression compared with PBS controls, whereas naïve B cell–derived PBs showed a similar but non-significant trend (Extended Data Fig. 5d and 5e). Both TGM1-hCD19 and hTGF-β1 treatments also significantly reduced Ki-67^+^c-Myc^+^ non-PB cells (Extended Data Fig. 5e). We next assessed the functional effects of TGM1-hCD19 on B cell antibody responses using the LAIV-stimulation assay in human spleen organoids (Fig. 3c). TGM1-hCD19 significantly suppressed LAIV-induced PB generation and reduced CD38 expression in the non-PB compartment (Fig. 3h–j), indicating B-cell silencing prior to PB differentiation^42^. Notably, hTGF-β1 failed to suppress PB generation in this system (Fig. 3h–j), likely due to pronounced “sink” effects from abundant non–B cell populations within organoids. Finally, TGM1-hCD19 significantly inhibited LAIV-induced influenza-specific IgG secretion compared with LAIV alone or combined with untargeted TGM1 or hCD19 scFv (Fig. 3k). In contrast, hTGF-β1 only modestly suppressed IgG secretion at day 4 had no significant effect by day 7 (Fig. 3k). Collectively, these results demonstrate that TGM1-hCD19 effectively silences antigen-driven B cell differentiation into PBs and IgG production in human spleen organoids.

## Human B-cell-targeted TGF-β agonist blocks B-cell differentiation trajectory toward antibody-secreting cells.

**Extended Data Figure 6.**
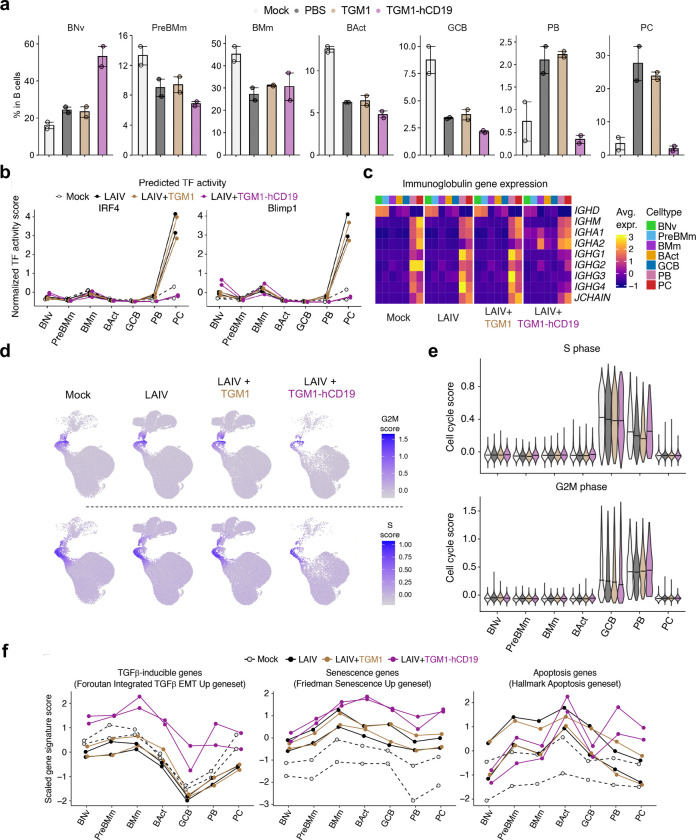
Suppressive effects of human B cell-targeted TGF-β agonist on antigen-driven B-cell differentiation in human spleen organoids **a.** Frequencies of immune cell subsets. **b.** SCENIC prediction of IRF4 and Blimp1 transcription factor activity. **c.** Immunoglobulin gene expression. **d.** UMAP plots showing cell cycle gene signature scores. **e.** Quantification of cell cycle gene signature scores. **f.** Scaled signature scores for TGF-β–inducible genes, senescence genes, and apoptosis-related genes, aggregated by sample.

**Figure 4.**
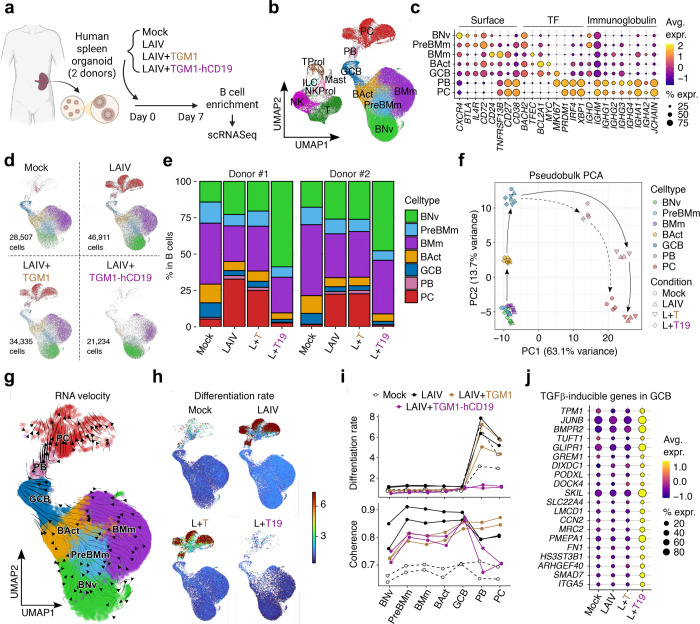
Human B cell-targeted TGF-β agonist suppresses antigen-driven B-cell differentiation in human spleen organoids. **a.** Experimental design for scRNA-seq sample preparation. **b.** UMAP visualization of all samples combined, colored by annotated cell types. **c.** Expression of B cell marker genes. **d.** UMAP visualization by treatment condition. **e.** Frequencies of B cell subsets. **f.** PCA plots. **g.** RNA velocity stream in mock-treated samples. **h.** Estimated rates of differentiation (i.e., cell state transitions) by treatment condition. **i.** Differentiation rates and coherence of velocity vectors. **j.** Leading-edge genes for the TGF-β pathway enriched in GCB cells treated with LAIV plus CD19-targeted TGM1.

To gain deeper insights into TGM1-hCD19-mediated suppression of antibody responses, we performed single-cell RNA sequencing (scRNA-seq) on B cells in spleen organoids of two donors (Fig. 4a). We performed unsupervised Louvain clustering on 163,632 cells surviving QC, with curated cell-type annotations transferred from a published human tonsil atlas^43^ (Fig. 4b). We focused on B-cell lineage cells (130,987 cells in total), identifying distinct clusters representing activated B cells (expressing *BCL2A1* and *MYC*), GCB cells (intermediate *CD38*, *BCL2A1*, and *MYC* with high *MKI67*), PBs (*MKI67*, *IGHM/A/G*, and *JCHAIN*) and plasma cells (PCs) (*IGHM/A/G* and *JCHAIN* without *MKI67*) (Fig. 4c). LAIV increased PBs by ~3-fold and PCs by ~10-fold compared with mock-treated organoids, while TGM1-hCD19 abolished this LAIV-driven increase of PB/PCs (Fig. 4d and 4e). Importantly, TGM1-hCD19 increased naïve B-cell frequency by 2~3-fold, while reducing activated B cells and GCB cells (Fig. 4e and Extended Data Fig. 6a), suggesting that B-cell-targeted TGF-β signaling actually rewires the entire B-cell population toward a naïve-like state rather than simply reducing antibody-secreting cells (ASCs). Pseudobulk aggregation followed by PCA revealed that TGM1-hCD19 did not appreciably shift transcriptional programs of non-PB/PC B cells (i.e., naïve to GCB cells) compared with mock, LAIV alone, and LAIV plus untargeted TGM1 (Fig. 4f). Conversely, PB/PC transcriptional programs differed between mock and LAIV-stimulated organoids, with TGM1-hCD19 rendering PB/PCs more similar to mock organoids (Fig. 4f). Consistently, gene-regulatory network analysis^44^ showed that TGM1-hCD19 reduced IRF4 and Blimp1 activity to baseline levels (Extended Data Fig. 6b). Finally, TGM1-hCD19 reduced *IGHG1/2/3/4* mRNA expression in PB/PCs to levels lower than mock, LAIV-, and LAIV plus untargeted TGM1-treated organoids (Extended Data Fig. 6c). Consistent with the literature reporting TGF-β-driven IgA expression, TGM1-hCD19 increased *IGHA1* and, notably, *IGHA2* mRNA levels in memory B cells and PB/PCs (Extended Data Fig. 6c), indicating that TGM1-hCD19 not only quantitatively reduces ASCs but also represses their IgG expression at the mRNA level.

**Extended Data Figure 7.**
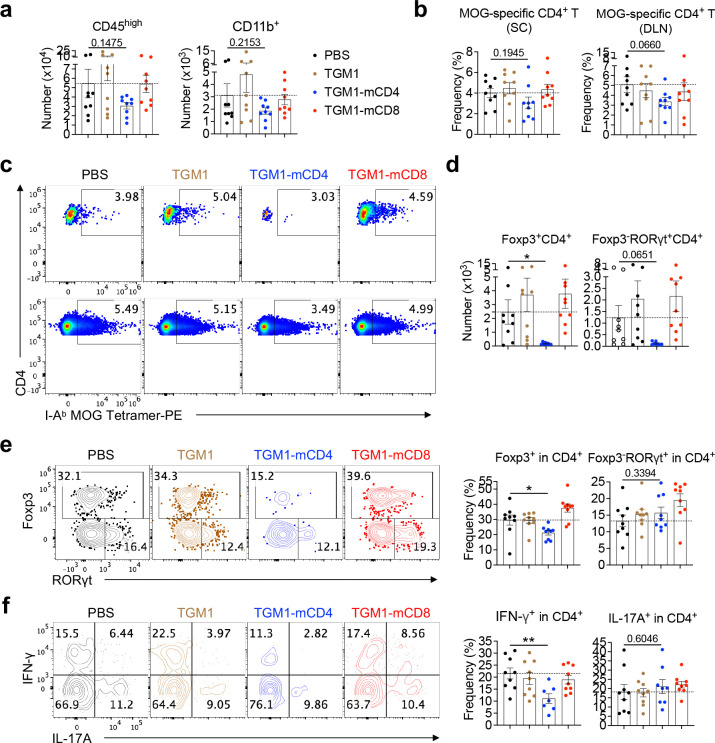
Therapeutic efficacy of the mouse T cell-targeted TGF-β agonist in EAE model. **a.** Quantification of CD45.2 and CD11b cell numbers in SCs of indicated mice. **b.** Quantification of MOG tetramer CD4 T cell frequencies in the SC. **c.** Representative flow cytometry plots showing MOG tetramer CD4 T cell frequencies in the SC. **d.** Quantification of Foxp3 and Foxp3 RORγt CD4 T cell numbers in the SC. **e.** Representative flow cytometry plots and quantification of Foxp3 and Foxp3 RORγt CD4 T cell frequencies in the SC. **f.** Representative flow cytometry plots showing IFN-γ– and IL-17A–producing CD4 T cell frequencies in the SC. Data are presented as mean ± s.e.m. The statistics were obtained by one-way ANOVA coupled with Dunnett’s multiple-comparisons test **(a, b, and d-f)**.

To understand the critical point where TGF-β signaling blocks B-cell differentiation toward PB/PCs, we next performed RNA velocity analysis^45^ (Fig. 4g). The RNA velocity vector field, embedded in the UMAP space, enabled inference of differentiation rates (i.e., the magnitude of dynamic change in the cell’s global mRNA transcription pattern) and coherence (i.e., concordance of the cell’s velocity vector with its neighbors) in each cell (Fig. 4h and 4i). The differentiation rates, which drastically increased at the PB stage in control organoids, remained nearly zero in TGM1-hCD19-treated organoids (Fig. 4h and 4i), indicating a profound silencing of GCB-to-PB differentiation. In the TGM1-hCD19-treated organoids, RNA velocity coherence remained high from naïve to GCB-cell stages but dropped at the PB stage (Fig. 4i), indicating a loss of differentiation “directionality” during the GCB-to-PB transition. This aligns with flow cytometry data showing that TGM1-hCD19 significantly reduced CD38 expression in the non-PB compartment (Fig. 3j), a key marker for human GCB cells. Pathway-specific analysis showed no substantial differences in S-phase and G2M-phase cell-cycle genes across conditions (Extended Data Figs. 7d and 7e); however, TGM1-hCD19 modestly increased senescence signature scores at later differentiation stages (Extended Data Fig. 7f). As expected, TGF-β signature scores were high across all B-cell subsets, including GCB cells, in the TGM1-hCD19-treated organoids (Fig. 4j and Extended Data Fig. 7f). Notably, GCB cells expressed those TGF-β signature genes at the lowest level compared to other B-cell subsets across conditions (Extended Data Fig. 7f), suggesting a critical role for the TGF-β signaling at this stage. Collectively, these observations suggest that B-cell-targeted TGF-β signaling imposes a qualitative phenotypic change at the GCB cell stage that abrogates their subsequent differentiation into ASCs.

## Therapeutic efficacy of mouse CD4 T cell-targeted TGF-β agonist in CD4 T cell–driven autoimmune neuroinflammation.

**Figure 5.**
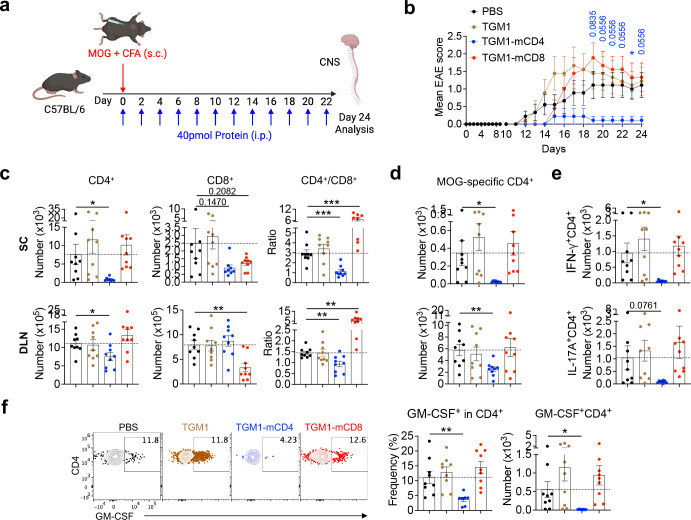
Therapeutic efficacy of mouse T cell-targeted TGF-β agonists in EAE model. **a.** Experimental design of MOG_35–55_ immunization and protein administration. **b.** Mean EAE disease scores of mice from the indicated groups. **c.** Quantification of CD4 and CD8 T cell numbers, and the CD4/CD8 T cell ratio, in spinal cords (SCs) and DLNs of indicated mice. **d.** Quantification of MOG tetramer CD4 T cells in SCs and DLNs of indicated mice. **e.** Quantification of IFN-γ– and IL-17A–producing CD4 T cells in SCs of indicated mice. **f.** Representative flow cytometry plots and quantification of GM-CSF–producing CD4 T cell frequencies and numbers in SCs of indicated mice. Data are presented as mean ± s.e.m. The statistics were obtained by two-way ANOVA coupled with Šídák’s multiple comparisons test **(b)** or one-way ANOVA coupled with Dunnett’s multiple-comparisons test **(c-f)**.

We next evaluated the therapeutic efficacy of T cell–targeted TGF-β agonists in disease models, first using an autoimmune neuroinflammation model of MOG – –induced experimental autoimmune encephalomyelitis (EAE) (Fig. 5a). The results showed that TGM1–mCD4 most effectively attenuated EAE development, whereas neither untargeted TGM1 nor TGM1–mCD8 provided significant therapeutic benefit (Fig. 5b), consistent with previous reports demonstrating that CD4 T cells are the primary drivers of this disease model^46^. Flow cytometric analysis further showed that TGM1-mCD4 treatment substantially reduced CD4 T cell numbers in both the spinal cord (SC) and DLNs, with CD45.2^high^, CD11b, and CD8 T cell numbers in the SC also trending downward (Fig. 5c and Extended Data Fig. 7a). In contrast, TGM1-mCD8 specifically reduced CD8 T cell numbers and increased the CD4/CD8 T cell ratio in the SC and DLNs (Fig. 5c). Notably, TGM1-mCD4 markedly decreased the absolute numbers of MOG-specific CD4 T cells in both SC and DLNs, despite minimal changes in their frequencies (Fig. 5d, Extended Data Fig. 7b and 7c). The frequency of Foxp3 Tregs in the SC was reduced, while RORγt cell proportions remained unchanged; however, the absolute numbers of both subsets were substantially decreased (Extended Data Fig. 7d and 7e). Similarly, the frequency of IFN-γ–producing CD4 T cells was reduced, and although IL-17A–producing cell frequency was largely unaffected, the absolute numbers of both populations were markedly diminished (Fig. 5e and Extended Data Fig. 7f). Importantly, both the frequency and number of GM-CSF–producing CD4 T cells in the SC were significantly reduced by TGM1-mCD4, indicating effective suppression of pathogenic Th17 cell development (Fig. 5f). These data demonstrate that TGM1-mCD4 effectively suppresses MOG-induced neuroinflammation by restraining inflammatory CD4 T cell differentiation and expansion.

## Therapeutic efficacy of T cell-targeted TGF-β agonists in mouse acute graft-versus-host disease models.

**Extended Data Figure 8.**
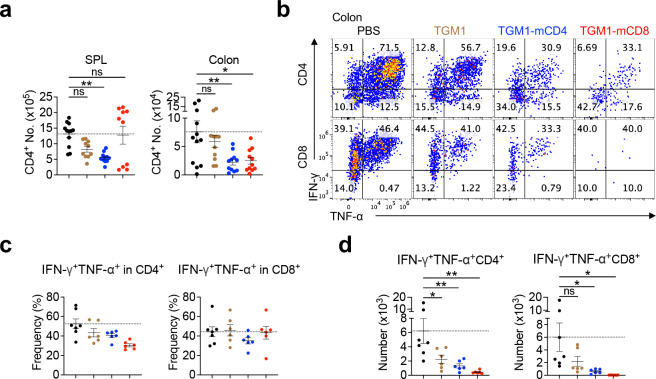
Therapeutic efficacy of T cell-targeted TGF-β agonists in GVHD model. **a.** Quantification of CD4 T cell numbers in spleens and colons. **b.** Representative flow cytometry plots showing IFN-γ and TNF-α production in colonic CD4^+^ or CD8^+^ T cells. **c.** Quantification of IFN-γ^+^TNF-α cell frequency among colonic CD4^+^ or CD8^+^ T cells. **d.** Quantification of IFN-γ^+^TNF-α CD4^+^ or CD8^+^ T cell numbers in the colon. Data are presented as mean ± s.e.m. The statistics were obtained by one-way ANOVA coupled with Dunnett’s multiple-comparisons test **(a, c, and d)**.

**Figure 6.**
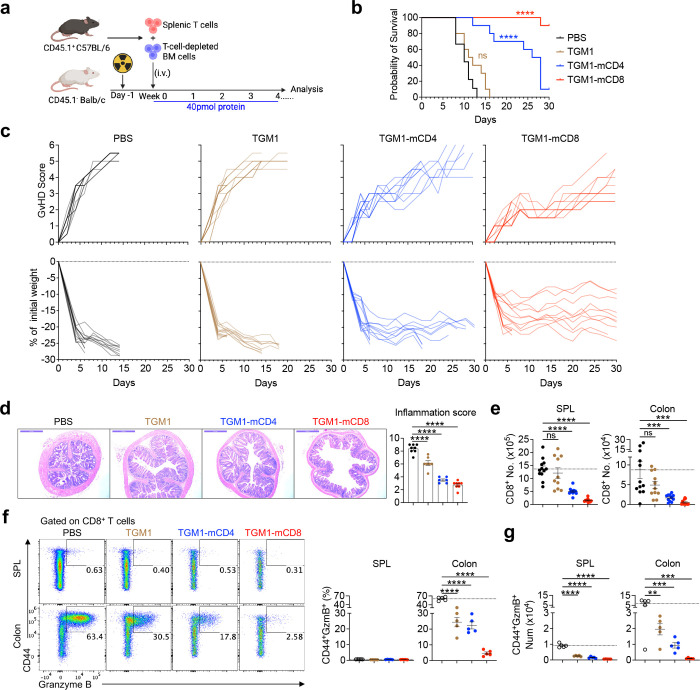
Therapeutic efficacy of mouse T cell-targeted TGF-β agonists in GVHD model. **a.** Experimental design of the mouse acute allogenic GVHD model and protein administration. **b.** Survival probability of mice in the indicated groups. **c.** Individual GVHD scores and body weight change curves of mice in the indicated groups. **d.** Representative histology images and quantification of colonic inflammation in the indicated groups. Scale bar, 1 mm. **e.** Quantification of CD8 T cell numbers in spleens and colons. **f.** Representative flow cytometry plots and quantification of CD44 Granzyme B cell frequencies among CD8 T cells in the colon from indicated groups. **g.** Quantification of CD44 Granzyme B CD8 T cell numbers in the colon from indicated groups. Data are presented as mean ± s.e.m. The statistics were obtained by log-rank (Mantel–Cox) test **(b)** or one-way ANOVA coupled with Dunnett’s multiple-comparisons test **(e-g)**.

We next evaluated the therapeutic efficacy of T cell–targeted TGF-β agonists in an acute allogeneic graft-versus-host disease (GVHD) model by adoptively transferring CD45.1 splenic T cells along with T cell–depleted bone marrow from C57BL/6 donors into lethally irradiated CD45.2 BALB/c recipients (Fig. 6a). Both TGM1–mCD8 and TGM1–mCD4 showed robust therapeutic efficacy in suppressing GVHD progression, as evidenced by improved survival, delayed and reduced GVHD scores, and reversal of body weight loss (Fig. 6b and 6c). In contrast, mice treated with PBS or untargeted TGM1 developed severe GVHD, with high clinical scores, pronounced weight loss, and mortality (Fig. 6b and 6c). As the gut is a major target organ of alloreactive T cells in this model, histological analysis showed that both TGM1-mCD8 and TGM1-mCD4 effectively suppressed colonic inflammation (Fig. 6d). Flow cytometric analysis showed that TGM1-mCD8 substantially reduced CD8 T cell numbers in both spleen and colon, whereas TGM1-mCD4 markedly reduced CD4 T cells and also partially decreased CD8 T cells (Fig. 6e and Extended Data Fig. 8a). Notably, colonic but not splenic CD8 T cells were highly activated and differentiated into cytotoxic Granzyme B–producing cells, and this population was strongly suppressed by TGM1-mCD8 and more modestly reduced by TGM1-mCD4 (Fig. 6f and 6g). These T cell–targeted TGM1 agonists also substantially reduced IFN-γ- and TNF-α-producing inflammatory T cell populations in the colon (Extended Data Fig. 8b–d). These data demonstrate that T cell–targeted TGM1 effectively suppresses mouse allogeneic GVHD, with both CD4 - and CD8-targeted TGM1 exhibiting significant therapeutic efficacy.

## Discussion

Here, we demonstrate that TGF-β agonism can achieve cell-specific silencing of antigen-driven immune responses in multiple mouse disease models and human spleen organoids. Unlike mAb- or CAR-T cell–mediated depletion therapies, our approach does not induce cytotoxic cell death and is therefore unlikely to trigger adverse events such as CRS or ICANS. Its high selectivity with minimal impact on non-target cells helps preserve overall immune homeostasis, in contrast to broader immunosuppressive agents like corticosteroids, tacrolimus, or anti-CD3 antibodies. Importantly, TGF-β signaling preferentially suppresses antigen-activated and proliferating T and B cells over resting cells, a pharmacological property that complements existing depletion therapies, which often spare the most pathogenic, activated, and proliferating immune cells.

While many T cell–potentiating agents are available for cancer therapy, therapeutically viable T cell–suppressing approaches remain scarce. Our CD4 and CD8 T cell–targeted silencing agents provide such an alternative, enabling both selective immunosuppression and dissection of the relative contributions of each subset in specific disease contexts. For instance, CD4 but not CD8 T cell–targeted TGF-β agonists conferred a clear advantage in the EAE model. The mouse CD4 T cell–targeted agonist further suppressed Tfh development, GCB cell formation, and antibody responses, while also limiting CD8 T cell expansion and effector functions (e.g., IFN-γ and granzyme B), indicating that CD4 T cell–targeted silencing can broadly modulate immunity in mice through both CD4 T–B and CD4 T–CD8 T cell interactions. In contrast, similar effects were not observed in an LAIV-stimulated human spleen organoid model, underscoring the greater complexity of human immune responses. Thus, directly targeting and silencing the disease-driving immune subsets may provide optimal immunosuppressive efficacy in human disease.

In both the OVA immunization and EAE models, CD4 T cell–targeted TGF-β agonists did not generate a substantial Treg population, despite partially inducing pTreg-like phenotypes. This is likely because strong TGF-β–mediated suppression of T cell expansion in the absence of IL-2 limits Treg proliferation, consistent with previous reports that conditional knockout of TβR1 in Tregs enhances their expansion and IL-10 production^47^. Thus, the therapeutic efficacy of our CD4 T cell–targeted TGF-β agonist is largely attributable to direct suppression of CD4 T cells rather than Treg induction. Moreover, the role of TGF-β in pathogenic Th17 cell development and disease progression remains controversial. Several studies have highlighted the Th17-promoting and pro-inflammatory effects of TGF-β signaling in T cells in EAE mouse models^48–50^, whereas other reports indicate that TGF-β–induced Th17 cells exhibit low pathogenicity and increased IL-10 production^51^. Our CD4 T cell–targeted TGF-β agonist, a gain-of-function approach, exhibited strong therapeutic efficacy in suppressing disease rather than exacerbating it, indicating that the overall CD4 T cell–suppressive effect outweighs any potential pro-inflammatory impact of the few Th17 cells induced by TGF-β signaling.

Anti-CD20 mAb-based B-cell depletion therapy has been in the clinic for over 20 years^6,7^, and CD19 CAR-T cell therapy, which can deplete CD20^−^ plasmablasts, has demonstrated higher efficacy in several trials for autoimmune diseases where anti-CD20 mAb therapy was ineffective^9–11^. Nevertheless, long-lasting plasma cells that lack CD19 expression persist and maintain preexisting humoral immunity to vaccine-related antigens even after CD19 CAR-T cell therapy, suggesting that even CD19 CAR-T cell therapy does not fully reset the humoral immune system. CD19-targeted TGF-β agonism, which acts primarily by silencing c-Myc^+^Ki67^+^ GC-like B cells poised to differentiate into PBs, represents an orthogonal approach to existing B-cell-depleting therapeutics. We also observed that this molecule increases naïve B cells while depleting more mature and activated B cells in the spleen organoids, a phenotypic shift concordant with the “reset” effect observed in the weeks after CD19 CAR-T cell therapy depletion in autoimmune patients^11^. Thus, the B-cell targeted TGF-β agonism depletes antibody-secreting cells without killing many B cells but instead limiting their maturation. Moreover, B-cell targeted TGF-β agonism has several advantages over existing B-cell-depletion therapies; first, B-cell-targeted TGF-β agonists may synergize with other intervention strategies, such as BTK inhibitors and our CD4^+^ T-cell-targeted TGF-β agonists; second, it does not require the harsh preconditioning regimens needed for CD19 CAR-T cell therapies, such as fludarabine or cyclophosphamide. The limitation of our B-cell data is that it relies on *in vitro* cultures and organoids. The relatively low affinity of the available targeting arms for mouse B cells (e.g., K_D_ = 42.7nM for the commonly used mouse CD19 scFv 1D3), unlike the human CD19 scFv FMC63 (K_D_ = 2.3nM)^41^, limits their utility for investigating the therapeutic efficacy of B-cell-targeted TGF-β agonism *in vivo* in mice. Nevertheless, the clear-cut phenotypes induced by the human CD19-targeted TGF-β agonist in complex systems such as spleen organoids provide proof of concept and support further mechanistic and therapeutic research.

Overall, this study provides proof of concept that targeted TGF-β agonists can precisely silence distinct immune cell types in human immune organoids and mouse disease models, highlighting cell-targeted TGF-β agonism as a versatile strategy for treating inflammatory, allergic, autoimmune, and transplant-related diseases.

## Methods

### Mice

Six- to ten-week-old female and male C57BL/6J (IMSR_JAX:000664) and BALB/c (IMSR_JAX:000651) mice, as well as other strains as indicated, were purchased from The Jackson Laboratory. OT-II (IMSR_JAX:004194) and OT-I (IMSR_JAX:003831) mice were crossed with Thy1.1 mice (IMSR_JAX:000406) to generate OT-II × Thy1.1 and OT-I × Thy1.1 mice. All animals were housed in AAALAC-accredited facilities. All experimental procedures were approved by the Stanford University Institutional Animal Care and Use Committee (IACUC; protocol IDs 32279, 34708, and 10269) and were conducted in accordance with institutional guidelines.

### Flow cytometry

The following antibodies were purchased from BioLegend: mouse CD45.2 (109839), CD3 (100206), CD4 (100453, 100430, 100428), CD8 (100706), NK1.1 (156506), TCRvα2 (127806, 127822), Thy1.1 (202528, 202522), CD25 (102012), CD44 (103026), CD69 (104543), CD11b (101259), GITR (126316), IL-17A (506928), IFN-γ (505832), Ki-67 (151212), TNF-α (506346), and IRF4 (646416); human CD3 (317324), CD4 (980806), CD25 (356134), FOXP3 (320126), CD86 (305432), IgM (314518), CD138 (356520), CD11c (337238), CD80 (305236), Ki-67 (350540), CD45 (304026), BCMA (357530), CD81 (349510), CD38 (303552), CD20 (302322), and HLA-DR (307674). The following antibodies were purchased from BD Biosciences: mouse Bcl6 (562401) and Rorγt (564722); human IgG (564229), CD21 (750614), Fas (752346), BAFFR (749942), CD10 (741825), CD23 (749448), IgD (562518), CD3 (560365), CD5 (566122), CD27 (566449), Bcl6 (569142), CD19 (555413), and Blimp1 (565274). The following antibodies and reagents were purchased from Invitrogen: mouse PD-1(48-9985-82), Foxp3 (12-5773-82, 17-5773-82), T-bet (25-5825-82), Fixable Viability Dye (65-0865-18). The following antibodies were purchased from Miltenyi Biotec: human IgA (130-113-480) and IRF4 (130-100-909). Ghost Red 780 Fixable viability dye was purchased from Cytek Biosciences (13-0865). Human c-Myc mAb was purchased from Santa Cruz Biosciences (sc-42 AF647). PE- and Brilliant Violet 421-labeled I-A^b^ MOG_38–49_ tetramers (GWYRSPFSRVVH) were provided by the NIH Tetramer Core Facility.

For cell-surface staining, live cells were incubated with antibodies and viability dye in PBS at 4°C for 1 h, and dead cells were excluded based on viability dye staining. For I-A^b^ MOG_38–49_ tetramer staining, live cells were incubated with tetramers in PBS at 37°C for 1 h, followed by surface antibody and viability dye staining. For transcription factor and cytokine staining, cells were fixed and permeabilized using the Foxp3/Transcription Factor Staining Buffer Set (00-5521-00, Invitrogen) and then stained intracellularly at room temperature for 2 h. For cytokine detection, cells were stimulated with Cell Stimulation Cocktail (00-4970-03, Invitrogen) and Protein Transport Inhibitor Cocktail (00-4980-03, Invitrogen) at 37°C for 5 h prior to fixation. Cells were resuspended in PBS and analyzed on a CytoFLEX flow cytometer (Beckman Coulter) or an Aurora cytometer (Cytek Biosciences); data were analyzed using FlowJo v10.10.0.

### Protein production

Recombinant proteins were cloned into the pD649 mammalian expression vector (ATUM), which encodes an HA secretion signal peptide, an N-terminal MSA fusion, and a C-terminal 6×His tag. Expression constructs were transfected into Expi293F^™^ cells using the Expi293^™^ Expression System (Thermo Fisher Scientific). After 3–4 days of culture, proteins were purified from culture supernatants by Ni-NTA agarose (Qiagen) and further purified by size-exclusion chromatography on a Superdex^™^ 200 column using an ÄKTA system (Cytiva). Endotoxin was removed using the Proteus NoEndo HC Spin Column Kit (VivaProducts) and verified to be at acceptable levels with the Pierce^™^ Chromogenic Endotoxin Quant Kit (Thermo Fisher Scientific). Final preparations were formulated and concentrated in sterile PBS, flash-frozen in liquid nitrogen, and stored at −80 °C until use.

### Mouse and human T cell isolation and *in vitro* culture

Mouse spleens and lymph nodes were harvested and mechanically dissociated to generate single-cell suspensions. Red blood cells were lysed with ACK lysis buffer (A10492-01, Gibco). CD4 or CD8 T cells were enriched by negative selection using the EasySep^™^ Mouse CD4 T Cell Isolation Kit (19852, STEMCELL Technologies) or Mouse CD8 T Cell Isolation Kit (19853, STEMCELL Technologies), respectively. Naïve T cells (CD44 CD25) were then sorted on a Sony SH800S cell sorter; sorted populations consistently exceeded 99% purity. Human CD4 or CD8 T cells were isolated from frozen PBMCs using the EasySep^™^ Human CD4 T Cell Isolation Kit (17952, STEMCELL Technologies) or Human CD8 T Cell Isolation Kit (17953, STEMCELL Technologies). Mouse and human T cells were cultured in RPMI 1640 with GlutaMAX^™^ (61870036, Gibco) supplemented with 10% FBS (A5256701, Gibco), 10 mM HEPES (15630080, Gibco), 1% sodium pyruvate (11360070, Gibco), 1% penicillin–streptomycin (15140122, Gibco), and 0.1% 2-mercaptoethanol (21985023, Gibco).

For mouse *in vitro* cultures, purified naïve T cells were plated at 0.75 × 10^6^ cells/mL in flat-bottom 96-well plates coated overnight with 5 μg/mL InVivoMAb anti-mouse CD3 (BE0002, Bio X Cell) and 2.5 μg/mL InVivoMAb anti-mouse CD28 (BE0015, Bio X Cell). Recombinant proteins were added at the indicated concentrations at culture initiation, and medium was refreshed with proteins on day 3. Cells were cultured at 37°C for a total of 5 days.

For human *in vitro* cultures, T cells were plated at 0.75 × 10^6^ cells/mL in flat-bottom 12-well plates coated overnight with 5 μg/mL InVivoMAb anti-human CD3 (BE0001, Bio X Cell) and 2.5 μg/mL InVivoMAb anti-human CD28 (BE0248, Bio X Cell) in the presence of 10 ng/mL recombinant human IL-2 (202-IL, R&D Systems) for 3 days. Cells were then re-plated at 0.75 × 10^6^ cells/mL in flat-bottom 96-well plates coated overnight with anti-human CD3 (5 μg/mL) and anti-human CD28 (2.5 μg/mL) and cultured with the indicated recombinant proteins. Medium was refreshed with proteins on day 3, and cells were cultured at 37°C for a total of 5 days.

### Mouse OVA immunization model

One million sorted Thy1.1 naïve OT-II and OT-I cells were mixed and adoptively transferred into Thy1.1 C57BL/6 recipient mice via retro-orbital intravenous injection. Starting 1 day post-transfer, mice were immunized with 100 μg OVA (A5503, Sigma-Aldrich) emulsified in complete Freund’s adjuvant (F5881-6X10ML, Sigma-Aldrich) and injected near the inguinal lymph nodes. The indicated proteins (40 pmol) were administered intraperitoneally every other day starting on day 0. Mice were euthanized on day 8 post-immunization, and inguinal lymph nodes and spleens were collected for analysis. For OT-II and OT-I bulk RNA-seq, T cells were enriched from inguinal lymph nodes on day 8, donor OT-II and OT-I cells were subsequently sorted, and samples were submitted to MedGenome, Inc. for library preparation and sequencing.

### Human spleen dissociation

Fresh spleen samples were obtained from deceased adult organ donors through Donor Network West (Northern California and Northern Nevada). The project was approved by the Donor Network West Ethics & Mission Committee. Spleens were decontaminated in Ham’s F-12 medium supplemented with Normocin and penicillin–streptomycin for at least 30 min at 4 °C. Tissues were transferred to Petri dishes, trimmed to remove large vessels, fat, and capsule, sectioned into ~5 g pieces, and mechanically dissociated into a paste. Each section was resuspended in 10 mL dissociation medium consisting of complete RPMI (RPMI 1640 with GlutaMAX, 10% FBS, 1× penicillin–streptomycin, 1× non-essential amino acids, 1× sodium pyruvate, 1× insulin–transferrin–selenium, and 1× Normocin) supplemented with 1 mg/mL collagenase IV and 200 U/mL DNase I. The slurry was transferred to gentleMACS C-tubes and processed using two rounds of dissociation on a gentleMACS Dissociator (protocol Spleen_04_01). Cell suspensions were filtered through a 100 μm strainer, resuspended in PBS containing 2% FBS and 6 mM EDTA, and washed once. Red blood cells were lysed with ACK lysis buffer for 5 min at room temperature, and cells were washed, counted, and aliquoted into 50 mL conical tubes. Granulocytes and residual erythrocytes were depleted using the EasySep Direct Human PBMC Isolation Kit (STEMCELL Technologies) according to the manufacturer’s instructions. Cells were then counted, resuspended in freezing medium (FBS with 10% DMSO), aliquoted into cryovials, frozen at −80 °C, and transferred to liquid nitrogen (−140 °C) for long-term storage.

### Human spleen organoid culture and stimulation

On the day organoid cultures were initiated, cryopreserved splenocytes were thawed in complete RPMI supplemented with 25 U/mL benzonase and incubated at 37 °C for 1 h. Organoid medium was prepared by supplementing complete RPMI with 1 μg/mL B-cell activating factor (BAFF). Twelve-well plates were set up with one 12-mm Transwell insert (0.4-μm pore size) per well, and 1 mL organoid medium was added to the lower chamber. Splenocytes were counted, washed, and resuspended at 6 × 10^7^ cells/mL. Aliquots of 100 μL splenocytes were transferred to 1.5-mL tubes, mixed with antigenic stimulus, and seeded into the Transwell inserts. For LAIV stimulation, 1 μL FluMist Quadrivalent (intranasal; 2022–2023 formulation) was added per well; mock controls received 1 μL PBS. On day 3, medium in the lower chamber was partially replaced.

### ELISA

For human ELISA to detect anti-influenza antibodies, Costar plates were coated with 0.1 μg per well of the 2022–2023 Fluzone Quadrivalent inactivated influenza vaccine (Sanofi) and incubated overnight at 4 °C. Plates were washed with PBS containing 0.05% Tween-20 (PBST) and blocked with PBS containing 1% BSA for 2 h at room temperature. Plates were then washed and incubated with spleen organoid supernatants for 1 h at room temperature, washed again, and incubated with horseradish peroxidase–conjugated anti-human IgG Fc secondary antibody (SouthernBiotech) for 1 h at room temperature. Signal was developed with 1-Step Ultra TMB substrate (Thermo Fisher Scientific, 37574), stopped with ELISA Stop Solution (Thermo Fisher Scientific, SS04), and absorbance was measured at 450 nm using a 96-well plate reader (Cytation 7, Agilent).

For mouse ELISAs, blood samples were centrifuged at 3,000 × g and serum was collected. OVA-specific IgG1 was quantified using a commercial ELISA kit (Mouse Anti-OVA IgG1 Antibody Assay Kit; Chondrex, 3013) according to the manufacturer’s instructions.

### Single-cell RNA-seq sample preparation

On day 7 of culture, human spleen organoids were dissociated by gently rinsing the Transwell membrane with FACS buffer (PBS supplemented with 0.5% BSA and 2 mM EDTA). B cells were enriched by negative selection using the Human Pan B Cell Isolation Kit (Miltenyi, 130-101-638) according to the manufacturer’s instructions. Cells were washed and resuspended in PBS containing 0.04% BSA. Single-cell capture was performed on a 10x Genomics Chromium X instrument, and gene expression libraries were generated using Single Cell 5′ R2-only v3 chemistry. Libraries were sequenced as paired-end 150-bp reads on an Illumina NovaSeq 6000. Reads were aligned to the GRCh38-2024-A reference transcriptome (including introns) and gene expression was quantified using Cell Ranger v9.0.1. Approximately 14,000-27,000 cells were recovered per sample.

### Mouse EAE model

Female mice were immunized subcutaneously with 100 μg MOG – peptide per mouse in complete Freund’s adjuvant (BD Biosciences) containing 200 μg Mycobacterium tuberculosis per mouse (BD Biosciences), injected at the axilla of both sides. Concurrently, 400 ng pertussis toxin per mouse (PTX, List Labs) was administered intraperitoneally, with a second dose given 48 hours later. The indicated proteins (40 pmol) were administered intraperitoneally every other day starting on day 0. Mouse body weight and clinical signs of disease were recorded daily and scored according to the following scale: 1 – tail paralyzed; 2 – hind limb weakness; 3 – one or both hind limbs paralyzed; 4 – front limb paralysis; 5 – moribund or deceased.

### Mouse allogeneic GVHD model

BALB/c female recipient mice received lethal total body irradiation (9 Gy). One day later, 5 × 10 T cell–depleted bone marrow cells and 1 × 10 splenic T cells isolated from donor female C57BL/6 mice were intravenously transferred into the BALB/c recipients by tail injection. The indicated proteins (40 pmol) were administered by intraperitoneal injection every other day starting on the day of cell transfer. Mice were monitored daily for clinical signs of GVHD, including weight loss, posture, mobility, and skin lesions. GVHD severity was scored according to standardized criteria (Extended Data Table 1). Mice were humanely euthanized if the GVHD score exceeded 6 or if body weight loss reached ≥30% relative to baseline. Flow cytometry and histological analyses were performed approximately 10 days after cell transfer.

### Isolation of immune cells from tissues

Mice were euthanized at the end of the indicated treatments. For lamina propria lymphocyte isolation, colons were opened longitudinally, cut into ~2 cm pieces, and incubated in 5 mM EDTA containing 1 mM DTT at 37 °C for 30 min to remove epithelial cells. Tissues were then minced and digested with DNase I (4 mg/mL; Roche) and collagenase D (0.5 mg/mL; Roche) at 37 °C for 30 min with shaking to generate single-cell suspensions, which were filtered through 70 μm strainers. For spinal cord lymphocyte isolation, mice were perfused, spinal cords were collected, mechanically dissociated using the plunger of a 1 mL syringe and filtered through 70 μm strainers to obtain single-cell suspensions. Lymphocytes were enriched using a 40%/70% Percoll gradient (Cytiva), and cells at the interface were collected for flow cytometry.

### Bulk RNA-seq analysis

Reads were aligned to the mouse reference genome (mm10) using Rsubread (v.2.18.0). Gene expression was quantified with featureCounts. For principal component analysis and heatmap visualization, variance stabilizing transformation (VST) was applied to the raw read counts. Differential expression (DE) analysis was performed with DESeq2^52^. Genes with FDR-adjusted P value < 0.05 and |log2FC| > 1 were considered DE unless otherwise noted. Gene Set Enrichment Analysis (GSEA) was performed against the Hallmark gene sets using log2FC-based gene ranking as input. For gene co-expression module analysis (also known as weighted gene co-expression network analysis; WGCNA), VST-transformed read counts were initially split into 45 gene modules, among which 14 modules were significantly enriched in TGM1-mCD4-treated CD4+ T cells or TGM1-mCD8-treated CD8+ T cells, when compared with other treatment conditions. Enrichment analysis was performed against TF perturbation genesets using the Enrichr webtool (https://maayanlab.cloud/Enrichr/). For celltype signature analysis, transcripts per million reads (TPMs) were calculated for all genes, and gene symbols were converted to corresponding human orthologs using the ENSEMBL repository. TPM values of sort-purified T-cell subsets were retrieved from the Monaco datasets available in the Human Protein Atlas (https://www.proteinatlas.org/humanproteome/single+cell/immune+cell/data#immune_cells_monaco). The TPM values were converted into log2 scale with the addition of the pseudocount of 1. We then applied the EPIC algorithm to the log-transformed TPM values to infer the composition of T-cell subsets in our bulk RNAseq data, using the Monaco dataset as a reference^53^. The predicted probabilities for each cell type were log-transformed and scaled, and used as a proxy of the proportion of adoptively transferred OT-I and OT-II T cells that have acquired the specific phenotype to construct radar charts.

### Single-cell RNA-seq analysis

The CellRanger-filtered gene expression matrix was first processed using Seurat (v.5.3.1). For each dataset, we excluded cells with fewer than 200 genes (to remove debris, empty droplets, and low-quality cells) and cells with higher than 9000 genes or higher than 100,000 reads mapped to RNA (to remove large cell aggregates). We also excluded cells in which >10% of transcripts were derived from mitochondrial RNA, leaving 163,632 cells in total (33,179, 51,696, 43,858, 34,899 cells in mock-, LAIV-, LAIV plus TGM1-, and LAIV plus TGM1-hCD19-treated groups, respectively).

We then performed a standard Seurat preprocessing workflow, including log-normalization, variable gene selection, scaling, PCA, and Uniform Manifold Approximation and Projection (UMAP) with 10 PCs using default parameters. To annotate cell types, we first performed unsupervised Louvain clustering on K-nearest-neighbor graphs (k = 20, resolution = 1). We applied the Seurat celltype-label transfer workflow using Human tonsil atlas data (ver2; https://zenodo.org/records/8373756) as the reference. The annotations stored in the “annotation_figure_1” slot were used for transfer. Transfer anchors were established in the 30-dimensional PCA space using genes in our data that were also present in the reference data. Seurat-defined clusters were manually annotated based on canonical marker gene expression and the aggregated prediction scores returned from the label transfer workflow.

For pseudobulk PCA, gene expression was aggregated by donor, condition, and cell type using the AggregateExpression function in Seurat. Transcription factor (TF) activity prediction was conducted on B-cell data (downsampled to 10,000 cells per condition) using the pySCENIC (v.0.12.1) Docker distribution with the default parameter settings. A total of 265 regulons representing unique TFs, including IRF4 and Blimp1, were identified. Normalized regulon activity scores were aggregated across donors, conditions, and cell types. Differential expression (DE) analysis was performed using Seurat’s FindMarkers function. Gene set enrichment analysis (GSEA) was performed using log2FC ranking as input against Hallmark and MSigDB C2 genesets (7,561 genesets in total) with the fgsea package. Leading-edge genes are selected for visualization as dot plots. For cell cycle gene signature scores, Seurats’s CellCycleScoring function was used with built-in S- and G2M-phase genes. For gene signature scores, Seurat’s AddModuleScore function was used. The scores were aggregated across donors, conditions, and cell types, and scaled for visualization.

For RNA velocity analysis, pre-mature (unspliced) and mature (spliced) transcript abundances were quantified with velocyto using position-sorted BAM files generated by the CellRanger pipeline, with filtering for cell barcodes that passed the Seurat QC step described above^45^. Outputs from velocyto were further analyzed using scVelo to estimate RNA velocity by stochastically modeling the transcriptional dynamics of splicing kinetics in individual cells^54^. The velocity vector fields were projected onto the UMAP space and visualized as velocity streams. The length of the velocity vector indicates the rate of differentiation (i.e., cell-state transition). The coherence of the vector field indicates how the differentiation pattern of a given cell correlates with that of its neighboring cells.

### Statistical analysis

Statistical analyses were performed using GraphPad Prism 10 (GraphPad Software). Data are presented as mean ± SEM, and P < 0.05 was considered statistically significant. Significance is denoted as *P < 0.05, **P < 0.01, ***P < 0.001, and ****P < 0.0001. Detailed statistical methods are provided in the corresponding figure legends. The number of mice per experiment is indicated by the dots in each figure.

## Extended Data

**Extended Data Table 1.** Assessment of clinical GVHD in transplanted mice.

Mouse allogenic GVHD model					
Criteria	Grade 0	Grade 0.5	Grade 1.0	Grade 1.5	Grade 2.0
Weight loss	<10%	N/A	10–24%	N/A	>25%
Posture	No hunch	Slight hunch, straightens when walks	Animal stays hunched when walks	Animal does not straighten out	Animal tends to stand on rear toes.
Mobility	Very mobile, hard to catch	Slower than naïve, easier to catch	Not moving, but will move when poked	Not moving, moves slightly when poked	Not moving, won’t move if poked
Skin	No redness, abrasions, lesions or scaling present	Redness in one area only	Abrasions in 1 area, or mild abrasions in 2 areas	Bad abrasions in 2 areas	Extremely bad abrasion, cracking skin, dried blood etc.

## Data Availability

The raw and processed bulk RNA-seq and scRNA-seq data generated in this study will be made publicly available in the GEO database upon manuscript acceptance. The R code used for RNA-seq data analysis will also be shared publicly at that time. Any additional information required to reanalyze the data reported in this paper is available from the lead contact upon request.
